# Editorial: Innate Immunity in Normal and Adverse Pregnancy

**DOI:** 10.3389/fimmu.2021.646596

**Published:** 2021-02-18

**Authors:** Richard M. Burwick, A. Inkeri Lokki, Sherry D. Fleming, Jean F. Regal

**Affiliations:** ^1^Division of Maternal Fetal Medicine, Department of Obstetrics and Gynecology, Cedars-Sinai Medical Center, Los Angeles, CA, United States; ^2^Bacteriology and Immunology, University of Helsinki, Helsinki, Finland; ^3^Research Programs' Unit, Translational Immunology Research Program, University of Helsinki, Helsinki, Finland; ^4^Obstetrics and Gynecology, Helsinki University Hospital, University of Helsinki, Helsinki, Finland; ^5^Division of Biology, Kansas State University, Manhattan, KS, United States; ^6^Department of Biomedical Sciences, University of Minnesota Medical School, Duluth, MN, United States

**Keywords:** innate immunity, complement, pregnancy, innate lymphoid cells, infection

In pregnancy, successful implantation, placental development and fetal growth, as well as maintenance of both maternal, and fetal health, requires balance of the immune response. Excessive activation of the immune system increases risk of rejection of the fetus and adverse pregnancy outcomes. In addition, disturbances in the immune system can lead to maternal or fetal infection. Due to cross-talk, both innate, and adaptive immunity must be properly regulated. Both our current topic “Innate Immunity in Normal and Adverse Pregnancy” and the parallel topic “Adaptive Immunity in Pregnancy” attracted 10–12 manuscripts. The articles in our topic included original research and comprehensive reviews and fell into 3 general categories: role of complement system, importance of innate immune cells, and an evaluation of innate immunity in infections in pregnancy. Each category evaluated the innate response in orchestrating a normal pregnancy or contributing to pathophysiology of adverse pregnancy outcomes (Figure 1). The timeliness of this topic is highlighted by the clear contribution of innate immunity to the pathology of COVID-19 infection and the potential impact on pregnancy outcomes (1, 2). This topic also synchronizes with the recent call by the Surgeon General in the United States indicating “we can—and must—do more for our moms” in reducing morbidity and mortality in pregnancy (https://www.hhs.gov/sites/default/files/call-to-action-maternal-health.pdf).

**Figure 1:**
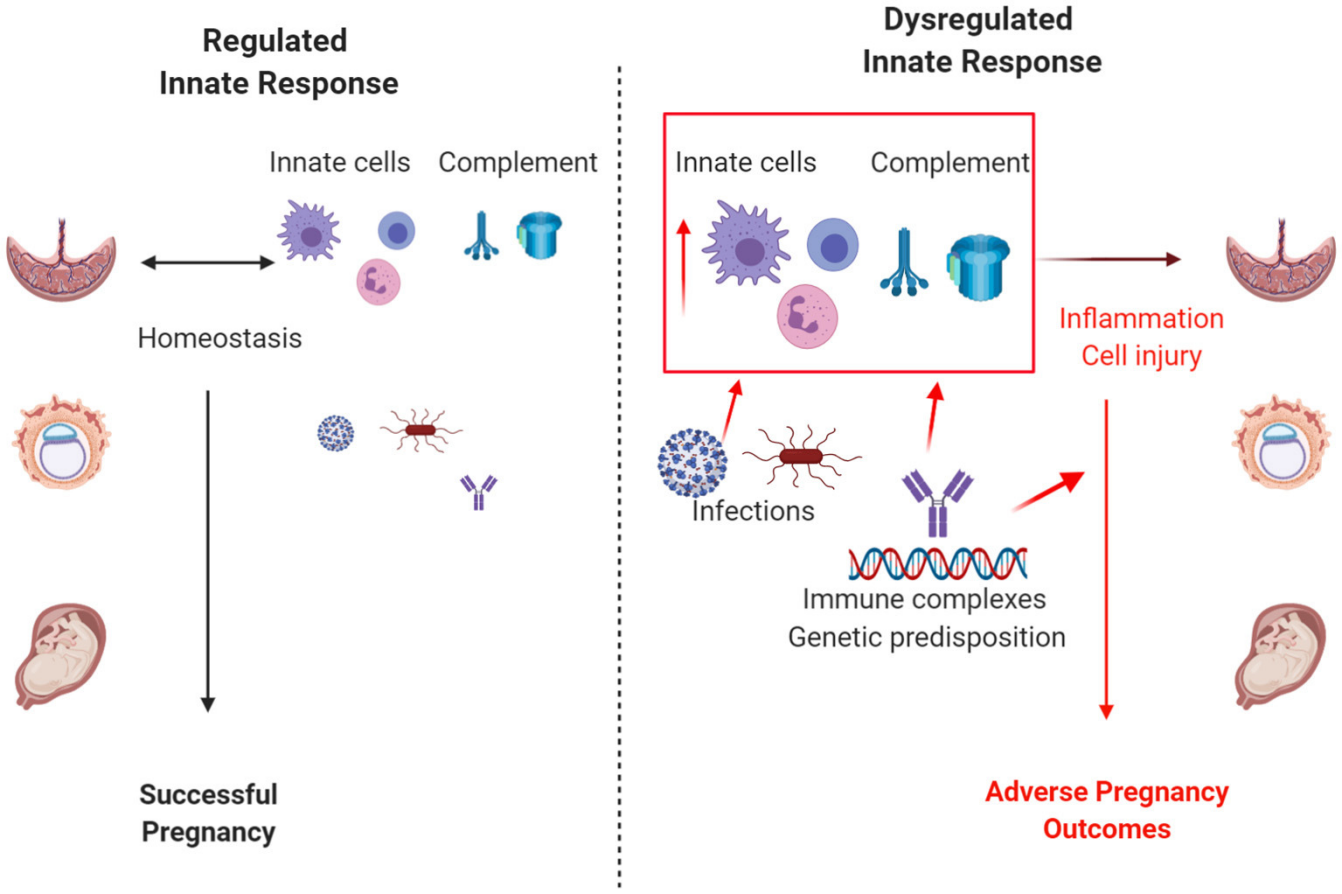
Regulation of the innate immune response influences pregnancy outcomes. When regulated, complement and the innate immune cells, including macrophages, neutrophils, and innate lymphoid cells, control infection and promote a successful pregnancy. Excessive or dysregulated innate responses due to uncontrolled infection, autoimmune disease, genetic predisposition, or other risk factors result in adverse pregnancy outcomes. Size depicts the magnitude of infection and/or activation of the innate immune response. Created with biorender.com.

## The Complement System In Normal and Adverse Pregnancy

The complement system is critical to healthy pregnancy due its central role in host defense. Activation of complement proteins provide immediate defense against foreign pathogens through opsonization, inflammation, and cell membrane attack. However, the role of complement in normal pregnancy extends well-beyond host defense. Girardi et al. provide an excellent review on the wide-ranging role of complement throughout pregnancy, including in normal pre-implantation, implantation, placental development, cervical remodeling, and parturition. To complete these normal pregnancy milestones successfully, homeostasis must be achieved by balancing regulation and activation of the complement system. Yet, we remain a long way from fully understanding how complement regulates even fundamental pregnancy processes, such as labor. Livson et al. shed some light on this topic, through original data demonstrating laboring women at term have greater C3 in cervicovaginal secretions, but a lower percentage of C3 activation, compared to non-laboring, or non-pregnant women.

Complement homeostasis is most critical at the maternal-fetal interface, where excess complement activation may lead to adverse pregnancy outcomes (3). Girardi et al. describe how the complement system becomes dysregulated at various stages leading to early pregnancy loss, placental insufficiency, preeclampsia and preterm birth, with associated long-term adverse effect to both mother and child. This association has been most well-characterized in pregnant women with immune complex disorders, such as systemic lupus erythematosus and antiphospholipid antibody syndrome (4). Chighizola et al. provide a current review describing the role of complement in pregnancy-associated autoimmune disease, expanding our understanding of the role of immune complexes and placental complement deposition in mediating adverse outcomes. Finally, Lokki et al. describe a case of pregnancy-associated atypical hemolytic uremic syndrome, which occurred after delivery for preeclampsia and HELLP syndrome. This case describes rapid clinical improvement and resolution of renal failure following complement (C5) blockade with eculizumab and emphasizes the emergence of complement inhibition as a therapeutic strategy for not only atypical hemolytic uremic syndrome, but potentially other pregnancy disorders characterized by complement dysregulation.

## Role of Innate Immune Cells In Pregnancy

The sentinel innate immune cells in decidua, placenta and fetus include neutrophils, macrophages, dendritic cells, innate B1 cells and innate lymphoid cells (5) (ILCs including NK cells). These innate immune cells protect both mother and fetus from infection throughout pregnancy and are also instrumental in orchestrating a normal pregnancy. Furthermore, these cells play a critical role in educating the adaptive response to induce tolerance. In general, the timeline of pregnancy is thought to cycle through an inflammatory phenotype in implantation to a protective anti-inflammatory state in maintenance of pregnancy, and a return to inflammatory phenotype at labor (6). However, Ono et al. present data in a mouse model indicating that while macrophages are required for implantation, the effective macrophages express an anti-inflammatory, M2-like phenotype. Thus, our simplistic view of inflammation in pregnancy requires modification. Similarly, dendritic cells are known to be essential for successful embryo implantation (7), and Yasuda et al. probes the phenotype of uterine dendritic cells between coitus and implantation to better understand their role. Mendes et al. reviews ILCs focusing on distinctions between peripheral and decidual NK cells. In addition, they highlight a potential role for other ILCs in establishing and maintaining a successful pregnancy and in contributing to adverse pregnancy outcomes. Much work is needed to fully understand the classification and contribution of Group 1, 2, and 3 ILCs and NK cells to pregnancy pathology.

During many pathophysiological conditions such as preeclampsia, both innate immune cells and barrier cells produce an exaggerated and inappropriate pro-inflammatory microenvironment. Aneman et al. discusses the cells contributing to the pro-inflammatory environment, including M1 macrophages, gamma delta T cells, NK cells and B1a B cells. Silva et al. demonstrates a contribution of decidual cells to the proinflammatory microenvironment with increased activation of NLRP3 inflammasome and subsequent IL-1β production in preeclamptic pregnancies without fetal growth restriction compared to normal pregnancies. Hypertensive pregnancy disorders, such as preeclampsia, result in increased risk of later-life cardiovascular disease for both the affected mother and the infant born from the preeclamptic pregnancy (8). Silva's data suggests that the potential benefits of statin treatment in pregnancy may be due to alterations in decidual cholesterol and the NLRP3 inflammasome, reducing risk of subsequent cardiovascular disease.

## Innate Immunity and Infection in Pregnancy

The innate immune system changes throughout pregnancy but these adaptations must not compromise the ability of the immune system to protect mother and child from infection. The innate immune response recognition of pathogen associated molecular patterns that interact with molecules such as Toll like receptors (TLRs) is an early line of defense for clearance of microorganisms. In pregnancy, bacterial infection has been associated with spontaneous preterm labor (9). However, the contribution of viral infection to preterm labor remains unclear. Rasheed et al. explore the concept of multi-pathogen induced preterm labor, revealing a synergistic effect of bacterial and viral TLR stimulation on a pro-inflammatory pro-labor response. These data lend support to the idea that multiple hits of viral and/or bacterial pathogens could increase the risk for preterm labor. These studies call for more detailed analyses of multiple pathogens both locally in the reproductive tract and systemically and their association with spontaneous preterm birth.

The review of innate immunity and viral infection during pregnancy by Cornish et al. provides an excellent overview of how acute viral infections challenge the critical balance between immune tolerance and defense against infection during pregnancy. The serious public health challenge posed by SARS-CoV2 infection and the concerns for upsetting a normal pregnancy demonstrates the timeliness of the topic. The fact that influenza and hepatitis E infection increase mortality of pregnant women, makes COVID-19 a clear cause for concern. Cornish et al. focuses on RNA viruses that may cause severe disease in pregnancy. The authors emphasize the importance of understanding the immune response during pregnancy and consideration of pregnancy in the design of vaccine trials. The review by Hoo et al. takes a slightly different view of innate immunity and infection, focusing on the human decidual placental interface during both bacterial and viral infections. How do infections take hold in the presence of this significant placental barrier? A review of the architecture and important innate immune cells is nicely diagramed to highlight both cellular and soluble immune defenses.

## Conclusion

This research topic highlights the diversity of innate immune processes crucial for a healthy pregnancy. More research is needed to enhance understanding of interaction and regulation of innate immunity during pregnancy and to introduce novel therapeutic strategies to address the clinical challenges posed by the complicated pregnancy. Thus, a healthy pregnancy requires an innate cellular and humoral response for proper development, but an inappropriate response results in adverse events.

## Disclosure

Opinions, interpretations, conclusions, and recommendations are those of the authors and are not necessarily endorsed by the American Heart Association, Department of Defense or National Institutes of Health.

## Author Contributions

All authors contributed to the writing of this editorial and approved the final submission.

## Conflict of Interest

Alexion Pharmaceuticals has supported speaking fees (AL and RB) and research grants (RB) in the past. The remaining authors declare that the research was conducted in the absence of any commercial or financial relationships that could be construed as a potential conflict of interest.

## References

[1] Makatsariya AD Slukhanchuk EV Bitsadze VO Khizroeva JKH Tretyakova MV Tsibizova VI. Thrombotic microangiopathy, DIC-syndrome and COVID-19: link with pregnancy prothrombotic state. J Matern Fetal Neonatal Med. (2020) 1–9. 10.1080/14767058.2020.1786811 32627622

[2] Rodrigues C Baia I Domingues R Barros H. Pregnancy and breastfeeding during COVID-19 pandemic: a systematic review of published pregnancy cases. Front Public Health. (2020) 8:558144. 10.3389/fpubh.2020.558144 33330308 PMC7719788

[3] Regal JF Gilbert JS Burwick RM. The complement system and adverse pregnancy outcomes. Mol Immunol. (2015) 67:56–70. 10.1016/j.molimm.2015.02.030 25802092 PMC4447554

[4] Kim MY Guerra MM Kaplowitz E Laskin CA Petri M Branch DW. Complement activation predicts adverse pregnancy outcome in patients with systemic lupus erythematosus and/or antiphospholipid antibodies. Ann Rheum Dis. (2018) 77:549–55. 10.1136/annrheumdis-2017-212224 29371202 PMC6037302

[5] Vivier E Artis D Colonna M Diefenbach A Di Santo JP Eberl G. Innate lymphoid cells: 10 years on. Cell. (2018) 174:1054–66. 10.1016/j.cell.2018.07.017 30142344

[6] Mor G Cardenas I Abrahams V Guller S. Inflammation and pregnancy: the role of the immune system at the implantation site. Ann N Y Acad Sci. (2011) 1221:80–7. 10.1111/j.1749-6632.2010.05938.x 21401634 PMC3078586

[7] Plaks V Birnberg T Berkutzki T Sela S BenYashar A Kalchenko V. Uterine DCs are crucial for decidua formation during embryo implantation in mice. J Clin Invest. (2008) 118:3954–65. 10.1172/JCI36682 19033665 PMC2582932

[8] Plummer MD Andraweera PH Garrett A Leemaqz S Wittwer M Aldridge E. Hypertensive disorders of pregnancy and later cardiovascular disease risk in mothers and children. J Dev Orig Health Dis. (2020) 1–6. 10.1017/S2040174420000896 33054877

[9] Minkoff H. Prematurity: infection as an etiologic factor. Obstet Gynecol. (1983) 62:137–44. 6346172

